# Regulatory function of the endogenous hormone in the germination process of quinoa seeds

**DOI:** 10.3389/fpls.2023.1322986

**Published:** 2024-01-08

**Authors:** Fang Zeng, Chunmei Zheng, Wenxuan Ge, Ya Gao, Xin Pan, Xueling Ye, Xiaoyong Wu, Yanxia Sun

**Affiliations:** Key Laboratory of Coarse Cereal Processing, Ministry of Agriculture and Rural Affairs, Sichuan Engineering and Technology Research Center of Coarse Cereal Industrialization, School of Food and Biological Engineering, Chengdu University, Chengdu, China

**Keywords:** *Chenopodium quinoa*, seed germination, plant endogenous hormones, RNA-seq, seed transcriptome

## Abstract

The economic and health significance of quinoa is steadily growing on a global scale. Nevertheless, the primary obstacle to achieving high yields in quinoa cultivation is pre-harvest sprouting (PHS), which is intricately linked to seed dormancy. However, there exists a dearth of research concerning the regulatory mechanisms governing PHS. The regulation of seed germination by various plant hormones has been extensively studied. Consequently, understanding the mechanisms underlying the role of endogenous hormones in the germination process of quinoa seeds and developing strategies to mitigate PHS in quinoa cultivation are of significant research importance. This study employed the HPLC-ESI-MS/MS internal standard and ELISA method to quantify 8 endogenous hormones. The investigation of gene expression changes before and after germination was conducted using RNA-seq analysis, leading to the discovery of 280 differentially expressed genes associated with the regulatory pathway of endogenous hormones. Additionally, a correlation analysis of 99 genes with significant differences identified 14 potential genes that may act as crucial “transportation hubs” in hormonal interactions. Through the performance of an analysis on the modifications in hormone composition and the expression of associated regulatory genes, we posit a prediction that implies the presence of a negative feedback regulatory mechanism of endogenous hormones during the germination of quinoa seeds. This mechanism is potentially influenced by the unique structure of quinoa seeds. To shed light on the involvement of endogenous hormones in the process of quinoa seed germination, we have established a regulatory network. This study aims to offer innovative perspectives on the breeding of quinoa varieties that exhibit resistance to PHS, as well as strategies for preventing PHS.

## 1 Introduction

Quinoa (*Chenopodium quinoa* Willd.), indigenous to the Andes region of South America, has emerged as a prominent food security crop in the 21st century (Vilcacundo and Hernández-Ledesma, 2017). Since the 1980s, quinoa has been introduced to numerous countries for cultivation due to its exceptional nutritional profile, earning it the title of “King of Grains” (Navruz-Varli and Sanlier, 2016). Quinoa, a stress-tolerant crop that flourishes in marginal soil and unstable climates, has emerged as a noteworthy agronomic plant choice (Nadali et al., 2020; Asher et al., 2022). Its increasing prominence in the global economy and the pursuit of a balanced diet can be attributed to the erratic climate patterns brought about by climate change and the growing emphasis on health (Jarvis et al., 2017). Quinoa yields in the United States and Canada vary between 840 and 2000 kilograms per hectare (750 to 1800 pounds per acre). However, the potential for complete yield loss exists due to pre-harvest sprouting (PHS) (Peterson and Murphy, 2015). Currently, the majority of research efforts on PHS have been directed towards understanding the dormancy and germination processes in other crops such as *Arabidopsis thaliana*, wheat, rice, and corn, with limited investigations on PHS in quinoa. It has been demonstrated that quinoa varieties exhibiting strong dormancy exhibit greater resistance to PHS, and promoting seed dormancy represents the most efficacious approach to suppressing PHS (Gallegos, 2022; Xu et al., 2022).

**Figure 1:**
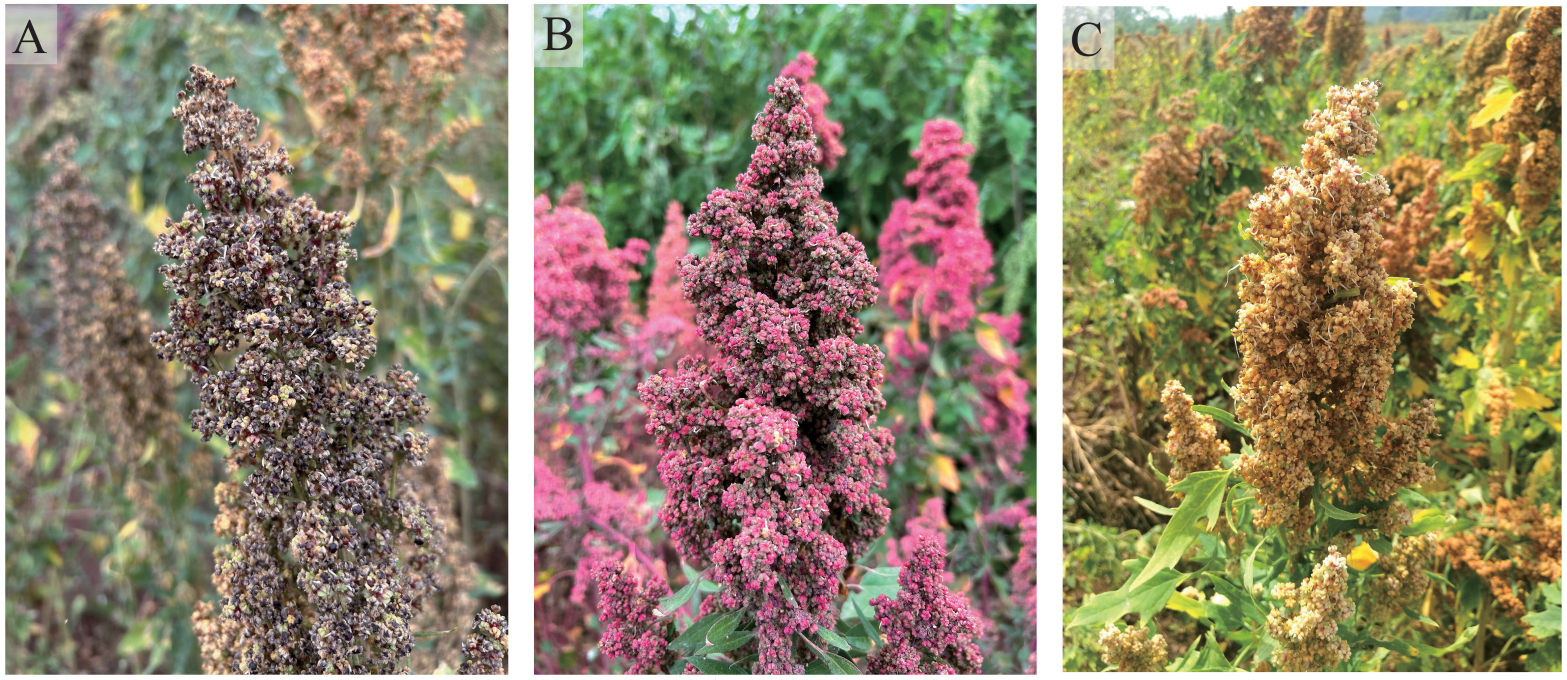
PHS of quinoa. **(A)** Cheng Li No. 2. **(B)** Cheng Li No. 1. **(C)** Long Li No. 1.

Abscisic acid (ABA) and gibberellin (GA) exhibit substantial antagonism in the regulation of seed germination (Barreto et al., 2020; Liu et al., 2020; Sohn et al., 2021; Tai et al., 2021). Through ongoing research on a range of endogenous hormones in plants, it has been revealed that auxin (IAA), jasmonic acid (JA), brassinosteroid (BR), ethylene (ETH), cytokinin (CKT), in addition to CKT and salicylic acid (SA), play a significant role in the regulation of seed germination (Miransari and Smith, 2014; Shu et al., 2016b; Ali et al., 2022). The relationship between IAA and JA with ABA in the regulation of seed dormancy has been extensively studied (Wang et al., 2011; Varshney and Majee, 2021). In a similar vein, it has been observed that ETH and BR exhibit a correlation with GA in facilitating the process of seed germination (Ahammed et al., 2020; Zhong et al., 2021). Nevertheless, the precise functions of CTK and SA in seed germination remain ambiguous.With the global population expanding and the frequency of natural disasters rising, there is a pressing need for a steady increase in crop yields to enhance people’s resilience to risks. PHS is one of the main factors leading to grain yield reduction (Sohn et al., 2021). Despite the global prevalence of PHS in regions where quinoa is cultivated (**Figure 1**), there remains a dearth of scholarly investigation on this matter, leading to an ambiguity in understanding the regulatory mechanisms of endogenous hormones in the manifestation of PHS in quinoa. This study employed quinoa seeds as the focal point of inquiry to assess the levels of α-amylase activity, soluble sugars, soluble protein, and starch throughout the process of germination. Furthermore, the quantification of eight endogenous hormones was accomplished using enzyme-linked immunosorbent assays (ELISA) and high-performance liquid chromatography electrospray ionization tandem mass spectrometry (HPLC-ESI-MS/MS) analysis. Following the completion of RNA-seq analysis, a total of 14 potential genes were identified as pivotal connectors within diverse hormone regulatory pathways. Ultimately, we established a comprehensive endogenous hormone regulatory network to elucidate the germination process of quinoa seed. This regulatory network differs significantly from that of common grain crops, which we speculate is due to the structural specificity of quinoa seeds. This study significantly enhances the comprehension of the underlying mechanisms pertaining to endogenous hormones in quinoa seed germination, while also offering valuable insights for the development of anti-PHS molecular breeding strategies and the resolution of PHS-related issues in quinoa.

## 2 Materials and methods

### 2.1 Plant material

The Quinoa seeds (Cheng Li No. 2) used in this study were provided by the Key Laboratory of Coarse Cereal Processing, Ministry of Agriculture and Rural, Chengdu University. This variety, which is high in protein content at 19.2% and was bred by Chengdu University, is highly susceptible to PHS (Pan et al., 2023b). Two sheets of filter paper were spread in a Petri dish with a diameter of 9 cm, and the sterilized quinoa seeds were placed in the Petri dish along with an appropriate amount of sterile water. The Petri dishes were then incubated in an artificial light-lit incubator at a temperature of (30.0 ± 1.0) °C, with a photoperiod of 16 hours of light and 8 hours of darkness, and a light intensity of 3000 Lux (Hao et al., 2022). The seeds were extracted from the incubator at specific time intervals (4 hours, 8 hours, 12 hours, 16 hours, and 20 hours). Subsequently, the seeds were drained with sterile filter paper to eliminate any surface moisture. The drained seeds were then transferred into 2 mL EP tubes and rapidly frozen with liquid nitrogen to prepare material for three biological replicate experiments. These frozen samples were subsequently stored in a -80°C refrigerator for further analysis.

### 2.2 Measurement of relevant indicators during quinoa seed germination

#### 2.2.1 Determination of germination rate and water absorption

A total of 100 sterilized quinoa seeds were carefully placed on sterile filter paper within a Petri dish. Subsequently, 8 mL of sterile water was added to the Petri dish, and germination experiments were conducted within an artificial climate chamber. Seed germination was observed at regular intervals of 4 hours within a 24-hour period, and the number of germinated seeds was documented. The criterion for germination was the breakthrough of the seed coat by at least 1 mm (Gao et al., 2023). A total of 1,000 seeds were selected and their mass was measured using an electronic balance. Subsequently, the seeds were placed in a petri dish of identical dimensions, immersed in water, agitated with a glass rod and let stand (Gao et al., 2023). The seeds were then removed from the dish at intervals of 4 hours, specifically at 4 hours, 8 hours, 12 hours, 16 hours, 20 hours, and 24 hours after water absorption. The surface moisture was removed by means of filter paper, and the corresponding weight was recorded for each time point. Germination rate (%) = number of germinated seeds/total number of seeds × 100%. Water absorption rate (%) = [(weight after immersion - weight before immersion)/weight before immersion] × 100% (Pan et al., 2023b).

#### 2.2.2 Methods for measuring physiological and biochemical indicators

The prepared plant material was removed from the refrigerator at -80°C for the determination of relevant indicators. The content of soluble protein, starch, and soluble sugar were determined in accordance with the instructions of the kit provided by Suzhou Keming Biotechnology Co., Ltd. The activity of α-amylase was determined according to the instructions of the kit provided by Shanghai Yuanye Bio-Technology Co., Ltd.

### 2.3 Determination of plant hormone content

The samples preserved at a temperature of -80°C were promptly pulverized into powder using liquid nitrogen, and the ABA, ZT (Zeatin), IAA, JA, GA_3_, and SA contents were assessed through the HPLC-ESI-MS/MS internal standard method (Niu et al., 2014; Liao et al., 2021). The specific techniques employed for measurement are provided in **Supplementary Text 1**. The content of ETH and BR was determined by Enzyme-linked Immunoassay Kit (Jiangsu Meimian industrial Co., Ltd) according to the manufacturer’s protocol.

### 2.4 Total RNA extraction and construction of a cDNA library

The total RNA extraction from each quinoa seed sample was performed using the RNA Easy Fast Plant Tissue Kit (Tiangen Biotech (Beijing) CO., LTD.) according to the manufacturer’s instructions, with three biological duplications. The concentration and purity of the RNA were assessed using the ScanDrop 200 (Analytik Jena AG, Germany). The integrity of the RNA was evaluated using the RNA Nano 6000 Assay Kit of the Agilent Bioanalyzer 2100 system (Agilent Technologies, CA, USA). Only RNA samples with a RIN (RNA integrity number) exceeding 8.0 were utilized for subsequent cDNA libraries construction. The initial step involved employing fragmented mRNA as a template and random oligonucleotides as primers to initiate the synthesis of the first strand of cDNA in the M-MuLV reverse transcriptase system. The RNA strand was degraded through the action of RNase H, and the second strand of cDNA was synthesized using the DNA polymerase I system, with dNTPs serving as the raw materials. The purified double-stranded cDNA then underwent end repair, followed by the addition of an adenylate tail and the attachment of a sequencing linker. Subsequently, cDNA fragments ranging from 250-300 bp were selected using AMPure XP magnetic beads. PCR amplification was then conducted, and the resulting PCR products were once again purified using AMPure XP magnetic beads. Ultimately, the cDNA library was obtained.

### 2.5 Illumina sequencing and data processing

After conducting a quality inspection of the library, Illumina sequencing was performed on the pooled libraries, which were selected based on optimal concentration and desired data volume. The raw data obtained from each sample was processed using the fastp software (Chen et al., 2018) followed by alignment of the clean data to the quinoa reference genome sequence (https://www.cbrc.kaust.edu.sa/chenopodiumdb) using the HISAT2 software (Kim et al., 2015). The transcript assembly for each sample was performed utilizing the StringTie software (Pertea et al., 2015), followed by merging the genome annotation of the assembled transcript for each sample. The GffCompare software (Pertea and Pertea, 2020) was employed to compare the annotations of all the sample transcripts with the reference genome annotation files. This enabled the extraction of unannotated transcripts from the reference genome and the identification of predicted new transcripts with a length of less than 100 and less than 2 exons. The gene expression levels were estimated by calculating the FPKM value of each gene utilizing StringTie (Pertea et al., 2015). The identification of differentially expressed genes (DEGs) was performed using the DESeq2 package in the R programming language (version 4.2.2), employing a model based on the negative binomial distribution (Love et al., 2014). The criteria for DEGs identification were set as |log2 (fold change)| > 1 and padj < 0.05.

### 2.6 Functional annotation and pathway enrichment analysis

To elucidate the function of the unigene, an extensive search was performed across various databases (Altschul et al., 1997), including Nr (NCBI non-redundant protein sequences), Swiss-Prot (Annotated Protein Sequence Database), KOG (Clusters of Protein homology), GO (Gene Ontology), and KEGG (Kyoto Encyclopedia of Genes and Genomes). Additionally, KEGG was utilized for further analysis of the biochemical pathways associated with the differential genes. The enrichment test and analysis of GO terms and KEGG pathways were conducted using the ClusterProfiler package of R language (v 4.2.2) (Yu et al., 2012).

### 2.7 qRT-PCR analysis

The quantification of endogenous hormone regulatory gene expression levels during the germination of quinoa seeds was conducted through the utilization of quantitative real-time polymerase chain reaction (qRT-PCR). The process involved the extraction and purification of RNA, followed by reverse transcription into complementary DNA (cDNA) using the FastKing RT Kit (With gDNase) FastKing cDNA (Tiangen Biotech (Beijing) CO., LTD.) as per the manufacturer’s instructions. The specific genes and corresponding primers employed in this study can be found in **Supplementary Table 1**. The qTOWER3 G Real-Time PCR System (Analytik Jena AG, Germany) was employed to perform the qRT-PCR reactions, which were replicated three times. The qRT-PCR program consisted of a pre-denaturation step at 95°C for 30 seconds, followed by 40 cycles of denaturation at 95°C for 5 seconds, and annealing/extension at 60°C for 30 seconds. The reference gene employed was Actin gene (ACT), and the relative gene expression levels were determined using the 2^−ΔΔCT^ method (Livak and Schmittgen, 2001).

### 2.8 Statistical analysis

The data collected in this study was organized using Excel 2016 software. Significance difference analysis was conducted using the one-way ANOVA and Tukey test methods of SPSS 19.0 software, with a significance level set at *P* < 0.05. The correlation analysis was performed using R language (v 4.2.2), and the resulting data was visualized using GraphPad Prism 9 and Cytoscape (v 3.7.1) software. All the experiments were repeated three times, and the results were calculated in x ± n. The RNA-seq data was submitted to the NCBI SRA database (accession number: PRJNA1028334).

## 3 Results

### 3.1 Dynamic changes in indicators related to quinoa seed germination

**Figure 2:**
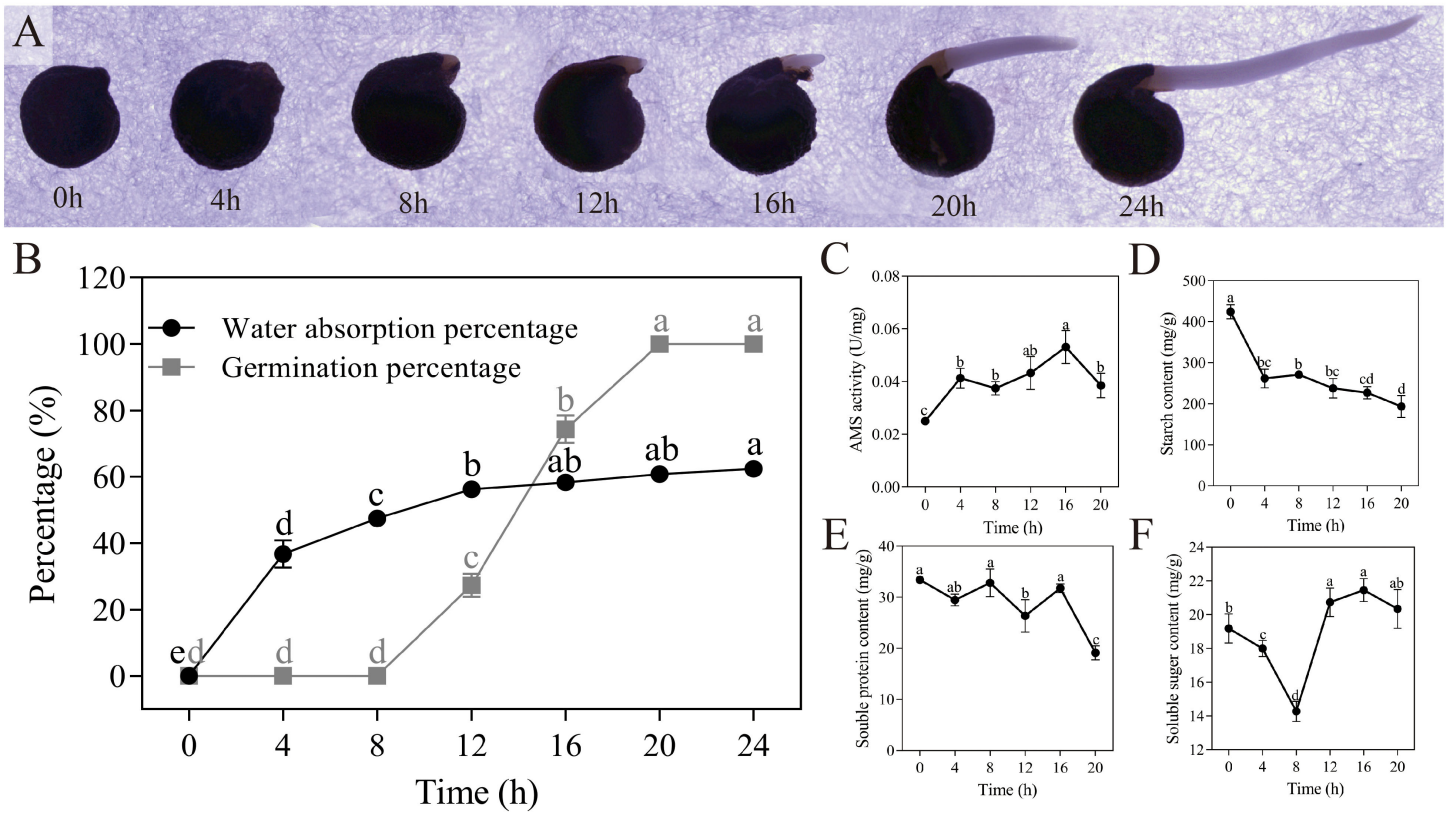
Dynamic changes in germination with quinoa seeds. **(A)** Progress of quinoa seed germination at 30°C from 0 hours to 24 hours. **(B-F)** Plots of water absorption and germination, α-amylase activity, starch content, soluble protein content, and soluble sugar content during germination of quinoa seeds. Different treatments marked with different lowercase letters showed significant difference (*P* < 0.05), while error bars represent the mean ± standard (n=3).

The morphology of quinoa seeds underwent changes during the germination, as depicted in **Figure 2A**. The rate of water absorption by the seeds exhibited a significant and rapid increase within the initial 8 hours (*P* < 0.05), reaching a rate of 47.45% at the 8-hour mark. Despite this heightened water absorption rate within 8 hours, the germination rate remained low at a mere 1/300. During the 8-20 hour period, the water absorption rate decelerated, while the germination rate experienced a significant increase (*P* < 0.05), ultimately reaching 100% at the 20-hour mark, as depicted in **Figure 2B**. Furthermore, the activity of α-amylase exhibited a consistent increase, with respective activities of 0.025 U/mg and 0.053 U/mg at 0 hours and 16 hours, as depicted in **Figure 2C**. This rise in α-amylase activity triggers the decomposition of starch within the seeds, resulting in a reduction in starch content (**Figure 2D**). Additionally, the total content of soluble protein displayed a decreasing trend, measuring 33.422 mg/g and 19.133 mg/g at 0 hours and 20 hours, respectively (**Figure 2E**). The soluble sugar content exhibited a decline followed by an increase, with contents of 14.279 mg/g and 21.455 mg/g at 8 hours and 16 hours, respectively (**Figure 2F**). Through data analysis, it is evident that quinoa seeds undergo an initial imbibition phase lasting from 0 to 8 hours, during which energy is accumulated for germination. Subsequently, the later stage of germination may involve a gradual balance between internal material synthesis and metabolism.

### 3.2 Changes in the endogenous hormone contents during seed germination

#### 3.2.1 Changes in GA_3_, ETH and BR content

**Figure 3:**
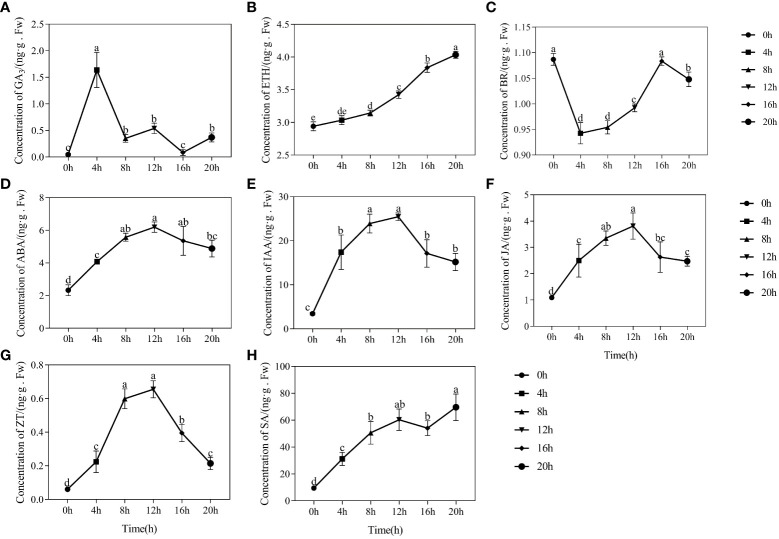
Changes in endogenous hormone content during the germination process of quinoa seeds. **(A-H)** represents the changes in endogenous hormone content of GA_3_, ETH, BR, ABA, IAA, JA, ZT, and SA during the germination process of quinoa seeds. Different treatments marked with different lowercase letters showed a significant difference (*P* < 0.05), while error bars represent the mean ± standard (n=3).

**Figure 3A** depicts a notable augmentation in GA_3_ content during the seed swelling stage, reaching a maximum of 1.638 ng/g at the 4-hour mark. Subsequently, the GA_3_ content gradually diminished, displaying a fluctuating pattern of change and an overall decrease in content. In contrast, the endogenous hormone ETH exhibited a consistent increase throughout seed germination, with contents of 2.941 ng/g at 0-hour and 4.034 ng/g at 20-hour (**Figure 3B**). The highest content of BR in dried seeds was determined to be 1.087 ng/g. Interestingly, it displayed a rapid decrease during the initial absorption phase of 0-4 hours, followed by a gradual increase. The BR content reached its lowest point at 0.942 ng/g at the 4-hour mark, but subsequently rose to 0.992 ng/g by the 12-hour mark (**Figure 3C**).

#### 3.2.2 Content changes in ABA, IAA, and JA

The alterations in ABA, IAA, and JA content during germination showed remarkable similarities, with a consistent pattern of initial increase followed by subsequent decrease (**Figures 3D-F**). The dried seeds had the lowest contents of ABA, IAA, and JA, measuring at 2.328 ng/g, 3.436 ng/g, and 1.090 ng/g, respectively. During the germination phase spanning of 8-20 hours, the contents of ABA, IAA, and JA experienced an initial rise before subsequently diminishing. At the 12-hour mark, the content of ABA, IAA, and JA peaked at 6.198 ng/g, 25.469 ng/g, and 3.812 ng/g, respectively. We found that there was a significant positive correlation between the decline in ABA, IAA, and JA levels and the enhanced seed germination rate.

#### 3.2.3 Content changes in CTK and SA

Prior to germination of quinoa seeds, there was a noticeable increase in the endogenous ZT content. Specifically, at the 8-hour and 12-hour, the ZT content reached 0.599 ng/g and 0.655 ng/g, respectively. Subsequently, as the process of seed germination progressed, there was a significant decrease in the ZT content, with a recorded value of 0.214 ng/g at the 20-hour mark (**Figure 3G**). In numerous investigations on stress resistance, it has been observed that SA has a stimulating effect on seed germination under stressful conditions (Lee and Park, 2014; Yan et al., 2023). Remarkably, our research demonstrates that the SA content tends to increase during the germination process (**Figure 3H**). Specifically, the SA content at 0 hours and 20 hours was measured at 9.445 ng/g and 69.592 ng/g, respectively, with a difference of 60.147 ng/g.

### 3.3 Illumina sequencing results and assembly

After the cDNA library has been constructed and assessed for quality, Illumina sequencing is conducted by combining various libraries based on the desired effective concentration and target data volume. The raw sequencing data for each sample is subjected to quality control using the fastp software (Chen et al., 2018). After the assembly procedure, a total of 37.10 G clean reads was obtained, demonstrating a GC content of 44.32% and a Q30 value of 92.80% (**Table 1**).

**Table 1:** Sequencing raw Data and Filtered data quality statistics.

Sample	Length	Reads	Bases	Q20 (%)	Q30 (%)	GC (%)
Sequencing raw Data quality statistics						
4h-1	150	45183914	6777587100	97.322	92.633	44.894
4h-2	150	42802694	6420404100	97.155	92.332	44.596
4h-3	150	43988100	6598215000	96.904	91.838	44.982
12h-1	150	42155318	6323297700	97.141	92.290	43.793
12h-2	150	42334706	6350205900	97.200	92.450	44.178
12h-3	150	42846718	6427007700	97.055	92.100	44.174
Filtered data quality statistics						
4h-1	143	44976220	6471473929	97.711	93.119	44.782
4h-2	143	42579486	6115274362	97.571	92.855	44.475
4h-3	143	43707740	6267867480	97.380	92.428	44.854
12h-1	144	41924370	6041239782	97.552	92.804	43.688
12h-2	144	42093168	6068680002	97.606	92.956	44.071
12h-3	144	42607348	6132486797	97.470	92.619	44.071

**Figure 4:**
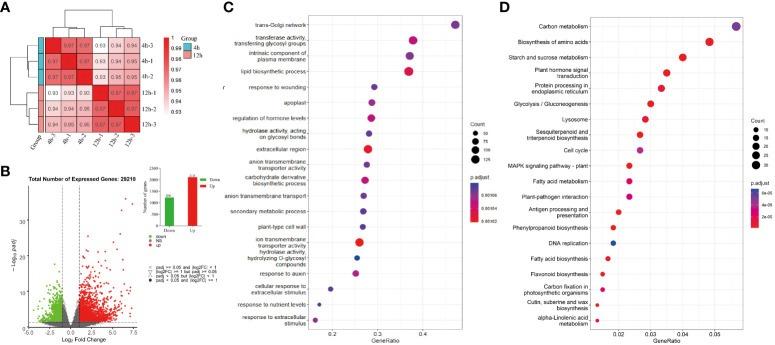
Correlation heat map, differential gene volcano map, and enrichment analysis map for each sample. **(A)** Pearson correlation coefficient of gene expression profiles between different groups of biological repeats, with group replicates exhibiting high correlation coefficients being grouped together. **(B)** Summarize the number of up-regulated and down-regulated gene sets based on 12-hour VS 4-hour differential gene sets. **(C, D)** GO molecular function and KEGG enrichment analysis.

The Pearson correlation coefficient was calculated to evaluate the correlation between different samples based on their gene expression levels. The resulting Pearson correlation coefficients for three biological replicates surpassed 0.933, indicating a robust association among these replicates (**Figure 4A**). A total of 29210 genes were found to be expressed in all samples. To identify genes with differential expression, a significance analysis was performed using the DESeq2 (Love et al., 2014) package, employing a screening criterion of |log2 (fold change)| > 1 and padj < 0.05. In our study, a total of 1236 downregulated differentially expressed genes and 2113 upregulated differentially expressed genes were identified (**Figure 4B**).

### 3.4 Unigene function annotation

The GO database annotation revealed that among the 29,210 single genes of quinoa, 952 GO terms were assigned, with 96 of them being associated with plant hormones (**Supplementary table 2**). Notably, the analysis revealed a significant enrichment of GO terms related to the regulation of hormone levels, with a considerable number of genes involved, as illustrated in **Figure 4C**. To further explore the involvement of hormone-related metabolic pathways in different stages of quinoa seed germination, the expected sequencing of individual gene biochemical pathways was subjected to additional analysis using the Kyoto Encyclopedia of Genes and Genomes (KEGG). A total of 22,503 individual genes were comprehensively annotated in the KEGG database and classified into 366 distinct signaling pathways (**Supplementary Table 3**).

**Figure 4D** visually represents the 20 biochemical pathways with the lowest padjs values for enrichment analysis. Among the KEGG enrichment pathways examined, the plant signal transduction pathways associated with the synthesis of plant hormones, specifically the ABA biosynthetic pathway (map ko00906), IAA biosynthesis pathway (map ko00380), GA biosynthetic pathway (map ko00904), ETH biosynthesis pathway (map ko00270), BR biosynthesis pathway (map ko00905), JA biosynthetic pathway (map ko00592), CKT biosynthesis pathway (map ko00908), and the SA biosynthetic pathway (map ko00360), exhibited significant enrichment. Therefore, the findings have demonstrated the significant involvement of plant endogenous hormones in the regulatory mechanisms governing the process of quinoa seed germination.

### 3.5 Identification of genes associated with the hormone regulatory network

**Figure 5:**
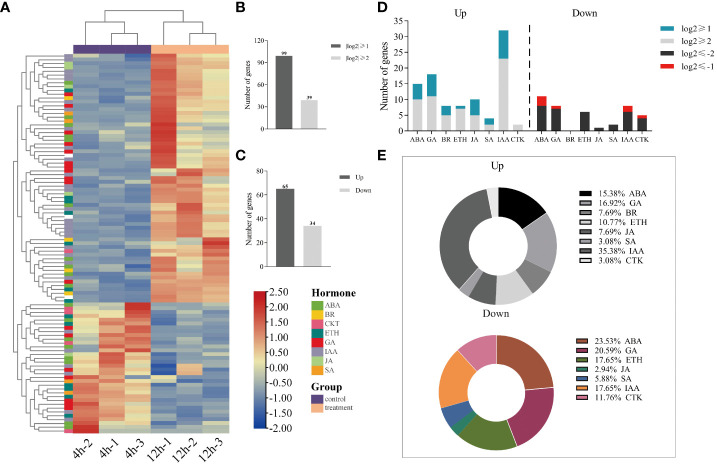
Analysis of differential expression genes involved in hormone regulation during the germination of quinoa seeds at 12 hours compared to 4 hours. **(A)** Heatmap of 99 differential gene expressions. **(B)** Histograms of 280 gene sequences with |log2 (fold change)| > 1 and |log2 (fold change)| > 2. **(C)** Histogram with up- regulation and down-regulation of expression in 99 differential genes. **(D)** Stacked histogram of the distribution of different genes involved in endogenous hormones. **(E)** Proportion of endogenous hormone-related differential genes.

A total of 280 gene sequences associated with the plant hormone pathway were identified through the application of transcriptome sequencing KEGG single hormone signaling pathway data. Out of these, 99 gene sequences exhibited a |log2 (fold change)| > 1, as indicated in **Supplementary Table 4**. Heat maps were generated to visualize the expression patterns of these genes, as shown in **Figure 5A**. Additionally, **Figure 5B** illustrates 39 gene sequences with a |log2 (fold change)| > 2. Among the 99 differentially expressed genes with a |log2 (fold change)| > 1, 65 genes exhibited increased expression while 34 genes displayed decreased expression, as depicted in **Figure 5C**.

As shown in **Figure 5D** and **Figure 5E**, a significant proportion of the differentially upregulated genes were found to be associated with ABA, GA, and IAA, accounting for 15.63%, 17.19%, and 35.94% respectively. Specifically, ABA and IAA exhibited 5 differential genes each, meeting the criterion of |log2 (fold change)| > 2, while GA had 7 differential genes satisfying the same condition. Among the down-regulated differential genes, ABA, GA, and IAA related genes accounted for a substantial proportion, representing 29.41%, 20.59%, and 17.65% respectively. We employed |log2 (fold change)| > 2 as the screening threshold to identify 10 ABA-related genes, 7-GA related genes, and 6-IAA related genes. This observation suggests that ABA, GA, and IAA may act as the principal regulatory hormones in the germination of quinoa seeds. Furthermore, the presence of other endogenous hormones in the regulation of differentially expressed genes indicates the involvement of multiple endogenous hormone-related genes in the germination process of quinoa seeds.

### 3.6 Quantitative analysis

**Figure 6:**
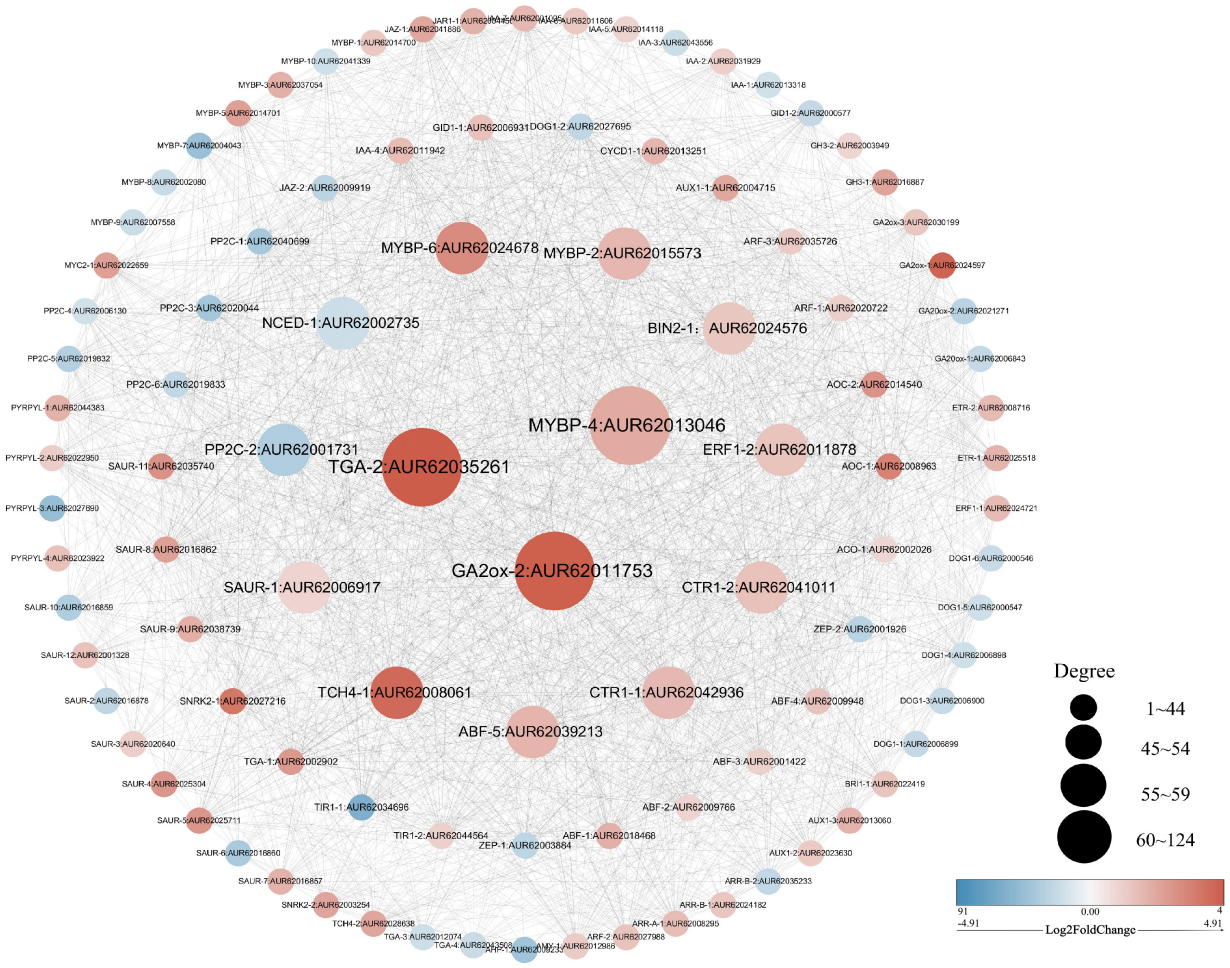
Correlation diagram of 99 related genes of hormone regulation in the seed germination pathway.

**Figure 7:**
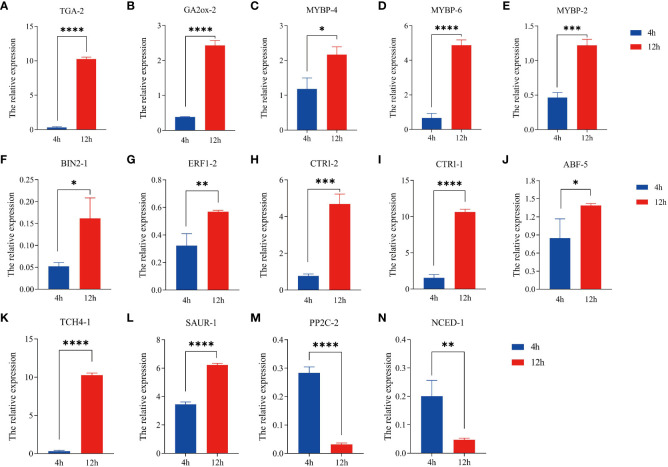
Expression profiles of hormone regulation-related genes in ‘Cheng Li No. 2’ seeds. **(A)** *TGA-2* (AUR62035261, transcription factor *TGA*). **(B)** *GA2ox-2* (AUR62011753, gibberellin 2beta-dioxygenase [EC:1.14.11.13]). **(C)** *MYBP-4* (AUR62013046, transcription factor *MYB*, plant). **(D)** *MYBP-6* (AUR62024678). **(E)** *MYBP-2* (AUR62015573). **(F)** *BIN2-1* (AUR62024576, glycogen synthase kinase 3 beta [EC:2.7.11.26]). **(G)** *EBF1-2* (AUR62011878, ethylene-responsive transcription factor 1). **(H)** *CTR1-2* (AUR62041011, serine/threonine-protein kinase CTR1 [EC:2.7.11.1]). **(I)** *CTR1-1* (AUR62042936). **(J)** *ABF-5* (AUR62039213, ABA responsive element binding factor). **(K)** *TCH4-1* (AUR62008061, xyloglucosyl transferase *TCH4* [EC:2.4.1.207]). **(L)** *SAUR-1* (AUR62006917, SAUR family protein). **(M)** *PP2C-2* (AUR62001731, protein phosphatase 2C [EC:3.1.3.16]). **(N)** *NCED-1* (AUR62002735, 9-cis-epoxycarotenoid dioxygenase [EC:1.13.11.51]). Data represent mean ± SD, n = 3. * *P* < 0.05, ** *P* < 0.01, *** *P* < 0.001, **** *P* < 0.001, vs. Control (4 hours).

The R.4.4.2 software was utilized to evaluate the correlation among 99 differential genes, leading to the discovery that a majority of these genes exhibited significant correlations (*P* < 0.05). Notably, 14 differential genes displayed degrees exceeding 55 (**Figure 6**). Given the process of quinoa seed germination, it is reasonable to speculate that these genes play crucial roles as central “transportation hubs” in hormonal interactions. Consequently, these 14 differentially expressed genes were chosen for qRT-PCR validation. The expression levels of the 14 genes examined exhibited statistically significant differences (*P* < 0.05) between the pre-germination (4 hours) and post-germination (12 hours) stages. These expression patterns were consistently in line with the findings from transcriptome sequencing, as depicted in **Figure 7**. Specifically, at the 12-hour stage, a noteworthy decrease in gene expression was observed for *NCED-1* and *PP2C-2* compared to the 4-hour stage. Conversely, the expression of the remaining 12 genes demonstrated a significant increase (*P* < 0.05). The gene expression level of *TGA-2*, *GA2ox-2*, *MYBP-6, CTR1-1, TCH4-1, SAUR-1*, and *PP2C-2* demonstrated a statistically significant augmentation at the 12-hour (*P* < 0.0001), with *TCH4-1* exhibiting a nearly tenfold increase.

## 4 Discussion

### 4.1 α-Amylase promotes starch hydrolysis to provide energy for seed germination

Along with the water absorption of seeds, the internal storage compounds, including starch and protein, are degraded by various hydrolases leads to the formation of sugars, thereby facilitating the development of the seed radicle and germ (Sano et al., 2022). The germination of quinoa seeds exhibited a consistent increase in α-amylase activity, which is in accordance with the observed pattern of α-amylase activity during the germination of African finger millet following water absorption (Gimbi and Kitabatake, 2009). The synthesis and release of α-amylase from the paste layer, induced by GAs, facilitates the conversion of starch molecules within the endosperm into readily sugars, thereby providing nourishment for the embryo’s growth (Brown et al., 2018). The findings of our study provide clear evidence of a strong correlation between the starch and α-amylase activity in quinoa seeds during the process of germination. Moreover, we observed a significant decrease in the starch content of quinoa seeds throughout germination, which can be attributed to the gradual hydrolysis of starch into glucose and other substances by amylase. We speculate that the initial decrease and subsequent increase in soluble sugar content in quinoa seeds during germination are also linked to this phenomenon. The findings are consistent with those of Anna-Sophie Hager (Hager et al., 2014). Additionally, our results indicate a general decline in the overall soluble protein content of quinoa seeds during the germination process. This phenomenon could potentially be attributed to the heightened enzymatic activity of proteolytic enzymes observed during the process of seed germination (Gupta et al., 2021).

### 4.2 Effects of plant endogenous hormones during quinoa seed germination

The intricate hormone regulatory networks observed in germinated plant seeds involve the antagonistic and synergistic interactions of endogenous hormones (Miransari and Smith, 2014; Shahzad et al., 2016). Previous research has indicated that GA_3_ undergoes a transformation from a bound to a free state and it plays a vital role in promoting seed germination (Li et al., 2019a). Our results showed that a significant increase (*P* < 0.05) in the endogenous GA_3_ content of quinoa seeds within the first four hours of water absorption, indicating the potential for substantial GA_3_ production during the initial stages of seed germination. The α-amylase activity and the soluble proteins content additionally confirmed the notion that GA_3_ mainly affects the germination of quinoa seeds in the initial stage of water absorption. ETH has also been reported to play a certain role in promoting seed germination (Ahammed et al., 2020). Our results demonstrated a general increase in ETH content during seeds germination, it is plausible to speculate that enhanced ETH synthesis may contribute to breaking the dormancy of quinoa seeds and subsequently facilitating germination. Exogenous ABA can inhibit the output of BR regulatory signals, consequently inhibiting BR-GAs-induced seed germination (Zhang et al., 2009; Xiong et al., 2021). We observed that the dried quinoa seeds exhibited a high content of BR and the BR content experienced a sharp decline to the lowest level 4 hours before seed germination, followed by a subsequent increase. Consequently, we speculated that the sudden reduction in BR content during the initial phase of quinoa seed germination may be attributed to the inhibition of BR synthesis by ABA, as well as the substantial depletion of stored BR during the seed swelling stage.

The ABA content typically decreases gradually during the germination process in seeds of sorghum, soybean, and rice (Benech Arnold et al., 2006; Gosparini et al., 2007; Xiao et al., 2018). However, our findings reveal a contrasting trend in quinoa seeds, where the ABA content increases during swelling, resembling the behavior observed in *Anemone rivularis* var. *flore-minore* and *desia polycarpa* Maxim seeds (Yanmei et al., 2018; Ge et al., 2020). Additionally, our results demonstrate that the contents of IAA and JA in quinoa seeds are consistent with those of ABA during the germination. This further verified the synergistic effect of IAA, JA, and ABA in inhibiting seed germination (Liu et al., 2013; Pan et al., 2020). The change in ABA content in *desia polycarpa* Maxim seeds during germination exhibited a similar trend to the findings of our study. However, the change in IAA and GA_3_ contents differed from our results (Yanmei et al., 2018). Our study suggests that the fluctuations in endogenous levels of IAA and JA during the germination process of quinoa seeds align with those observed in rice seeds, while the alterations in ABA content exhibit inconsistency (Xiao et al., 2018). The hormone content during the process of germination can differ among seeds of different species, and different quinoa varieties may exhibit either primary or physiological dormancy, or may not exhibit any dormancy at all (Baskin and Baskin, 2007; McGinty et al., 2021). In order to enhance resilience to particular natural calamities, it is plausible that a feedback regulatory mechanism exists for the negative regulation of germination hormones within quinoa seeds, thereby augmenting their synthesis during the initial phases of germination and impeding the process.

Our study showed a significant positive correlation (*P* < 0.01) between ZT content and ABA during quinoa seed germination, and SA content exhibited the highest throughout all time periods of quinoa seed germination. The roles of ZT and SA in the regulation of seed germination have been a subject of controversy (Miransari and Smith, 2014; Anaya et al., 2018; Tuan et al., 2019; Yan et al., 2023). Previous research has suggested that SA can impede seed germination by inhibiting the activity of α-amylase induced by GAs in the cells of the pasteurized layer. Conversely, SA has also been found to promote seeds germination under stressful conditions (Xie et al., 2007; Lee and Park, 2014). Based our research findings, we propose a research question: is there a correlation between the tolerance of quinoa towards environmental stress and the internal regulatory function of SA?

### 4.3 Hormone related regulatory factors in the germination process of quinoa

GA counteracts the inhibitory impact of the DELLA protein on seed germination by binding to the plant gibberellin receptor GID1 and degrading DELLA (Yoshida and Ueguchi-Tanaka, 2014). The results of our study showed that the expression of *DELLA* (*1-2*) and *GID1-2* during the process of quinoa seed germination is concomitant with an upregulation in the expression of *GID1-1*. This suggests that *GID1-1* within the gene *GID1* (*1-2*) may play a major role in regulating quinoa germination. In contrast, in rice, gibberellic acid triggers seed germination by activating the transcription factor *GAMYB*, which in turn influences amylase (AMY) activity (Washio, 2003). Notably, only *MYBP-4* and *MYBP-6* in quinoa seeds showed a significant positive correlation with *AMY-1* (*P* < 0.05). Therefore, we speculated that the transcription factors *MYBP-4* and *MYBP-6* could interact with *AMY* to promote the germination of quinoa seeds. To our surprise, a notable decrease in the expression of the GA20ox regulatory gene *GA20ox* (*1-2*) was observed during quinoa seed germination, whereas a significant increase in the expression of the GA2ox regulatory gene *GA2ox* (*1-2*) was observed (*P* < 0.05). *GA20ox1* and *GA2ox9* are responsible for governing the synthesis and metabolism of GA within seeds, thereby exerting control over seed germination and dormancy (Fukazawa et al., 2021; Xing et al., 2023). Concurrently, ABA triggers the transcription of the GA 2-oxidase 7 gene (*GA2ox7*) and stabilizes the ABI4 protein, leading to GA degradation and the induction of seed dormancy (Shu et al., 2016a). These findings imply that the regulatory mechanisms governing GA synthesis and degradation during quinoa seed germination may exhibit species-specific characteristics. In relation to BR, it was observed that the expression level of the regulatory genes *BIN2-1* (Brassinosteroid insensitive 2, BIN2) and *BRI1-1* (Brassinosteroid insensitive 1, BRI1) were significantly higher at the 12-hour. The correlation coefficients between *BRI1-1* and the quinoa GA synthesis gene *GA20ox-1* were -0.886, while the coefficients between *BRI1-1* and the GA catabolism genes *GA2ox-1* and *GA2ox-2* were 1 and 0.880, respectively. These findings suggest that BR may also affect the *ABF* genes during quinoa seed germination through the involvement of the two regulators, *BIN2-1* and *BRI1-1*. These regulators influence the functioning of *ABF* genes and promote GA synthesis via BRI1, thereby promoting seed germination. This result is consistent with the reported conclusion that BR can promote seed germination by attenuating ABA on seed germination and promoting GA_3_ synthesis (Hu and Yu, 2014; Zhao et al., 2018; Kim et al., 2019; Zhong et al., 2021). We also found that the BR-regulated pathway effector *TCH4-1* (Xyloglucosyl transferase) was significantly increased nearly 9-fold in expression during germination of quinoa (*P* < 0.0001), but its specific regulatory role needs to be investigated in depth. The regulatory pathways of ABA and GA were observed to interact with the ETH pathway, leading to an elevation in ETH production, which was correlated gradual accumulation of ACC-synthase (ACS) transcripts and the activity of ACC-oxidase (ACO) (Wang et al., 2005; Rzewuski and Sauter, 2008; Corbineau et al., 2014). With the germination of quinoa seeds in our study, the expression level of gene *ACO-1* was also extremely significantly increased (*P* < 0.0001). This discovery has led us to suggest a plausible correlation between the heightened endogenous ETH content and the increased expression of *ACO-1* genes. In *Arabidopsis thaliana*, it has been observed that ethylene response factor 12 (ERF12) could bind to the promoter region of the dormant key gene delay of germination 1 (*DOG1*), thereby exerting inhibitory effects on the expression of *DOG1*. This interaction, known as “Ethylene response 1 (ETR1)*-*ERF12*-*Topless (*TPL*)*-DOG1*”, has been identified as a negative regulator of the seed dormancy pathway (Li et al., 2019b). ETR1 has been also found to engage in co-regulation of ethylene’s signaling pathway through its interaction with the Constitutive triple response 1 (CTR1) (Ju et al., 2012). In this study, a comprehensive analysis was conducted on 10 differential sequences linked to the pathway under investigation in quinoa seeds. The expression levels of *ETR* (*1-2*), *CTR1* (*1-2*), and *ERF1* (*1-2*), found to be significantly augmented, while the genes associated with *DOG1* (*1-6*) exhibited reduced expression. Our findings suggest the existence of a potential negative regulation of quinoa seed dormancy, referred to as “ETR-CTR1-ERF-*DOG1*”, which exhibits similarities to the germination process of *Arabidopsis thaliana*.

ABA receptors (PYR/PYL/RCAR) interact with and suppress the activity of PP2C (Protein phosphatase 2C, a negative regulator of ABA signaling), leading to the activation of Sucrose non-fermenting 1-related protein kinases (SnRKs). This activation subsequently enhances the regulatory effect of downstream ABRE-binding protein (AREB)/ABRE-binding Factor (*ABF*) transcription factors, which ultimately inhibits seed germination (Carrera-Castano et al., 2020). Overexpression of 9-cis-epoxycarotenoid dioxygenase 6 (NCED6) in seeds results in an increased ABA content during the imbibition period, consequently promoting seed dormancy (Martínez-Andújar et al., 2011). In our study, we observed a down-regulation of the gene *NCED-1* during the germination of quinoa seeds, which may be attributed to the short dormancy period of quinoa (McGinty et al., 2021). Additionally, we found significant increases (*P* < 0.05) in the differential sequences of three key components involved in the ABA early sensing and signaling pathway: *SNRK2* (*1-2*), *PYR/PYL* (*1-2*), and *PYR/PYL-4*. Conversely, we noted significant decreases (*P* < 0.05) in the expression of *PYR/PYL-2* and *PP2C* (*1-6*). Therefore, we speculated that the regulatory mechanism of ABA in quinoa seed germination is species-specific. IAA is another endogenous plant hormone, apart from ABA, that has been recently discovered to induce seed dormancy. IAA can indirectly stimulate the expression of abscisic acid insensitive 3 (*ABI3*) through ARF10 and ARF16, which are auxin responsible factors, ARFs (Liu et al., 2013). Additionally, the ARF2 mutant *arf2* exhibited heightened sensitivity to ABA during seed germination, providing further evidence for the regulatory role of ARF2 in ABA signaling. This regulation is achieved through the interaction of ARF2 with its target gene *HB33*, ultimately influencing seed germination (Wang et al., 2011). During the process of quinoa seed germination, a notable positive correlation (*P* < 0.01) was observed between the *ARF* (*1-3*) genes and the *ABF* (*1-5*) genes. Furthermore, the expression levels of these genes exhibited a significant increase (*P* < 0.05) upon seed germination. The inhibitory effect of ABA on seed germination is weakened by the auxin signaling inhibitor Aux/IAA8 through the suppression of *ABI3* expression (Hussain et al., 2020). Our study reveals that a decrease in *TIR1-1* (Transport inhibitor response 1) expression leads to an increase in *IAA-2* and *IAA (4-7)* expression, which may enhance the inhibitory influence on the “ARF-ABF” pathway. Both ABA and JA can inhibit seed germination, and their interconnection can be facilitated by the involvement of PYL6 (RCAR9) and *MYC2* (Aleman et al., 2016; Pan et al., 2023a). During the process of quinoa germination, we found 15 distinct genes associated with JA regulation of seed germination, and among them, 6 genes exhibited significant differences (*P* < 0.05). Notably, the correlation coefficients between *PYR/PYL-1* and *PYR/PYL-2*, as well as *MYC2-1*, were found to be 0.829. This suggests that the interaction between ABA and JA in quinoa may also be mediated through the participation of PYL6 and *MYC2*. The JA signaling receptor F-box protein coronatine insensitive 1 (COI1) initially forms a complex called SCF^COI1^ (Skp1/Cullin1/F-box protein COI1) by binding to the proteins SKP1 and Cullin1. This complex then interacts with the JAZ inhibitor, leading to the degradation of the JAZ1 protein and ubiquitination of the 26S proteasome. Consequently, downstream JA transcription factors are activated, enabling the regulation of JA response events (Devoto et al., 2002; Pan et al., 2020). The JAZ protein, in turn, inhibits the transcriptional activity of *ABI3* and abscisic acid insensitive 5 (*ABI5*). However, the introduction of exogenous ABA can degrade the JAZ protein and facilitate seed germination (Ju et al., 2019; Pan et al., 2020). During the process of quinoa germination, our study revealed a significant increase in the upregulation of *JAZ-1* (*P* < 0.0001) and *ABF1-5* (*P* < 0.05). Additionally, a negative correlation was observed between the decrease in *JAZ-2* expression and *ABF1-5* (*P* < 0.05). Meanwhile, the expression levels of the positive regulators *JAR1-1* and *COI1-1*, which are involved in the JA regulatory pathway, was also found to be enhanced. Moreover, the regulatory signals of ABA exhibited the potential to amplify the functionality of the allene oxide cyclase (AOC) in the JA synthesis pathway (Wang et al., 2020). Therefore, we postulated that the significant increase of *AOC* (*1-2*) (*P* < 0.05) expression in quinoa resulted in the increase of endogenous JA content during quinoa seed germination, consequently impeding the germination process.

**Figure 8:**
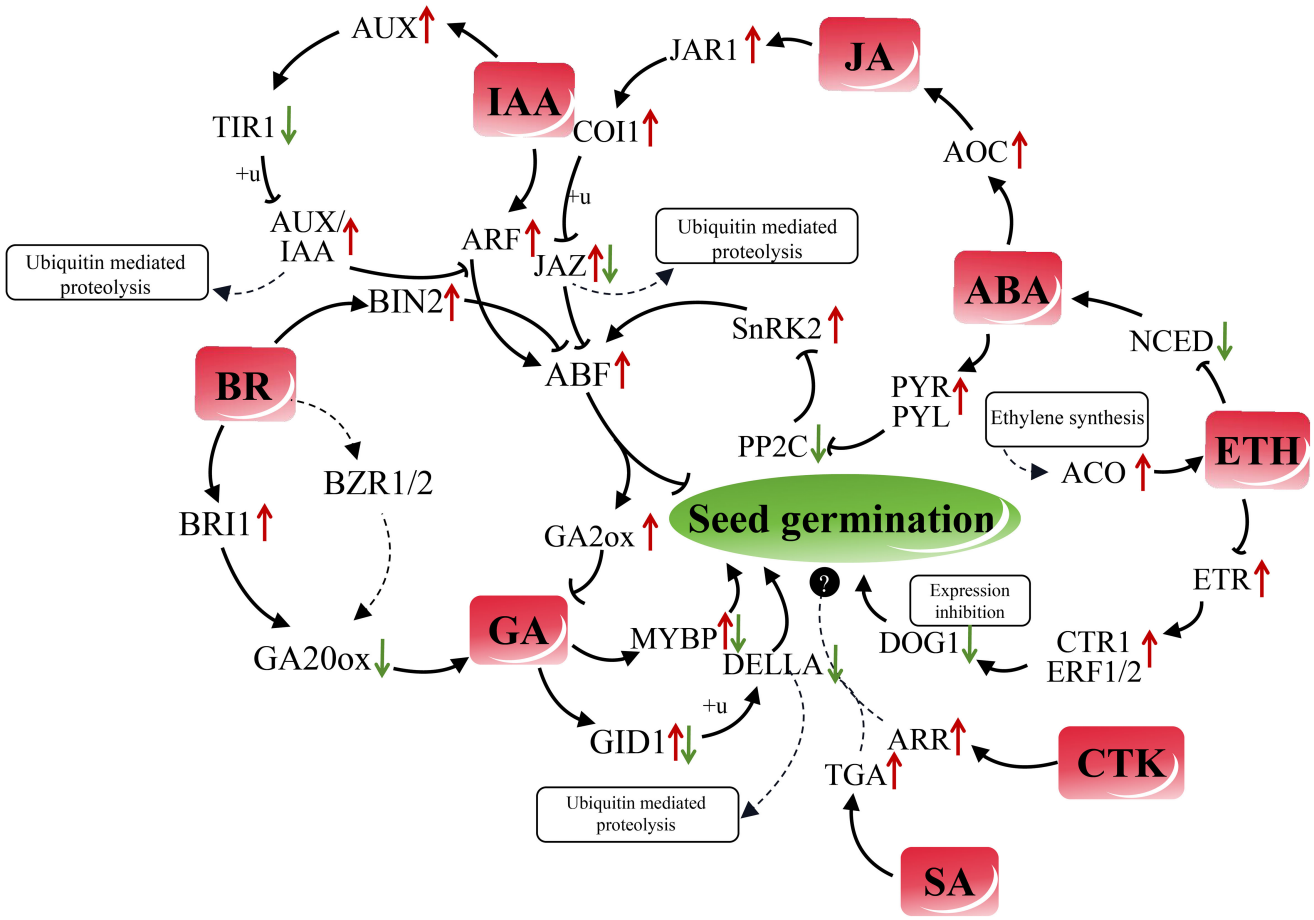
Regulatory network of plant hormone functions during the germination process of quinoa seeds. ABA receptors (PYR/PYL/RCAR) bind to and inhibit the activity of PP2C, leading to the activation of SnRKs and subsequent increase the expression of downstream transcription factor *ABF*, thereby inhibits seed germination. The up-regulation of AOC by ABA promotes the increase of synthesis of JA, and the expression of JA receptors JAR1 and COI1 is upregulated as a result. These receptors, in turn, impact the regulatory genes of JAZ protein, and the degree of ubiquitination of JAZ protein influences its inhibitory effect on *ABF* gene expression. The upregulation of the IAA response gene *ARF* has the potential to impact the expression of the *ABF* gene. Simultaneously, the expression of the auxin influx facilitator AUXI is enhanced, while the expression of *TIR1* downstream is reduced. *TIR1* plays a role in ubiquitinating AUX/IAA protein, thereby reducing the inhibitory effect of AUX/IAA on the “ARF-*ABF*” interaction. In the BR signaling regulatory pathway, the GSK3 kinase BIN2 negatively regulates *ABF*, leading to a diminished effect of *ABF* on the GA2ox decomposition regulatory gene. Additionally, the BR receptor kinase BRI1 regulatory gene influences the content of GA through *GA20ox*. Further research is required to confirm the ability of the transcription factor *BZR1/2* to bind to the promoters of *GA20ox1* and other GA synthesis genes, thereby inducing GA expression. GA plays a crucial role in seed germination by regulating the expression of the receptor gene *GID1*, leading to the ubiquitination of DELLA protein. Besides, GA promotes seed germination by activating the transcription factor regulatory gene *MYBP*. Furthermore, the synthesis of ethylene promotes the upregulation of ACO expression in the ethylene synthesis pathway, specifically ACC-oxidase, resulting in an increase in ETH content. ETH, in turn, inhibits the expression of the ABA synthesis gene *NCED*. ETH has a negative regulatory effect on the expression of its receptor gene *ETR*, leading to the promotion of CTR1 and *ERF1/2* expression, inhibition of the dormant regulatory gene *DOG1* expression, and subsequent facilitation of seed germination. The potential positive regulatory roles of genes *ARR* in the CTK regulatory pathway and genes *TGA* in the SA regulatory pathway in quinoa seed germination require additional verification. Black arrows denote positive regulation, while bars represent negative regulation. Red and green arrows are employed to signify increased and decreased gene expression, respectively.

Through an examination of the expression patterns of CKT-related genes, namely *TacZOG*, *TaGLU*, and *TaARR12*, in embryonic tissues of both dormant and non-dormant genotypes, it was determined that CKT possesses the ability to regulate seed dormancy (Tuan et al., 2019). In our study, the expression levels of the *ARR-A-1* and *ARR-B-1* genes, which are link to CKT, exhibited a significant increase (*P* < 0.05) during quinoa seed germination, indicating that ARR may serve as a positive regulator in this process. SA also has a significant effect on seed germination, but the regulatory pathways of seed dormancy and germination have not been thoroughly studied (Anaya et al., 2018; Yan et al., 2023). And to our surprise, the expression of TGA-2 in the SA-regulated pathway exhibited a substantial ten-fold increase (*P* < 0.0001) following quinoa germination. Quinoa, being a quintessential example of a crop resilient to environmental stress, holds significant promise for research in the regulation of seed germination by SA. As a typical representative crop of tolerant to environmental stress, SA regulation of quinoa seed germination has great research potential. Drawing upon the aforementioned regulatory genes and alterations in hormone content, a comprehensive network of endogenous hormones during the 4-12 hour period of quinoa seed germination was established (**Figure 8**).

**Figure 9:**
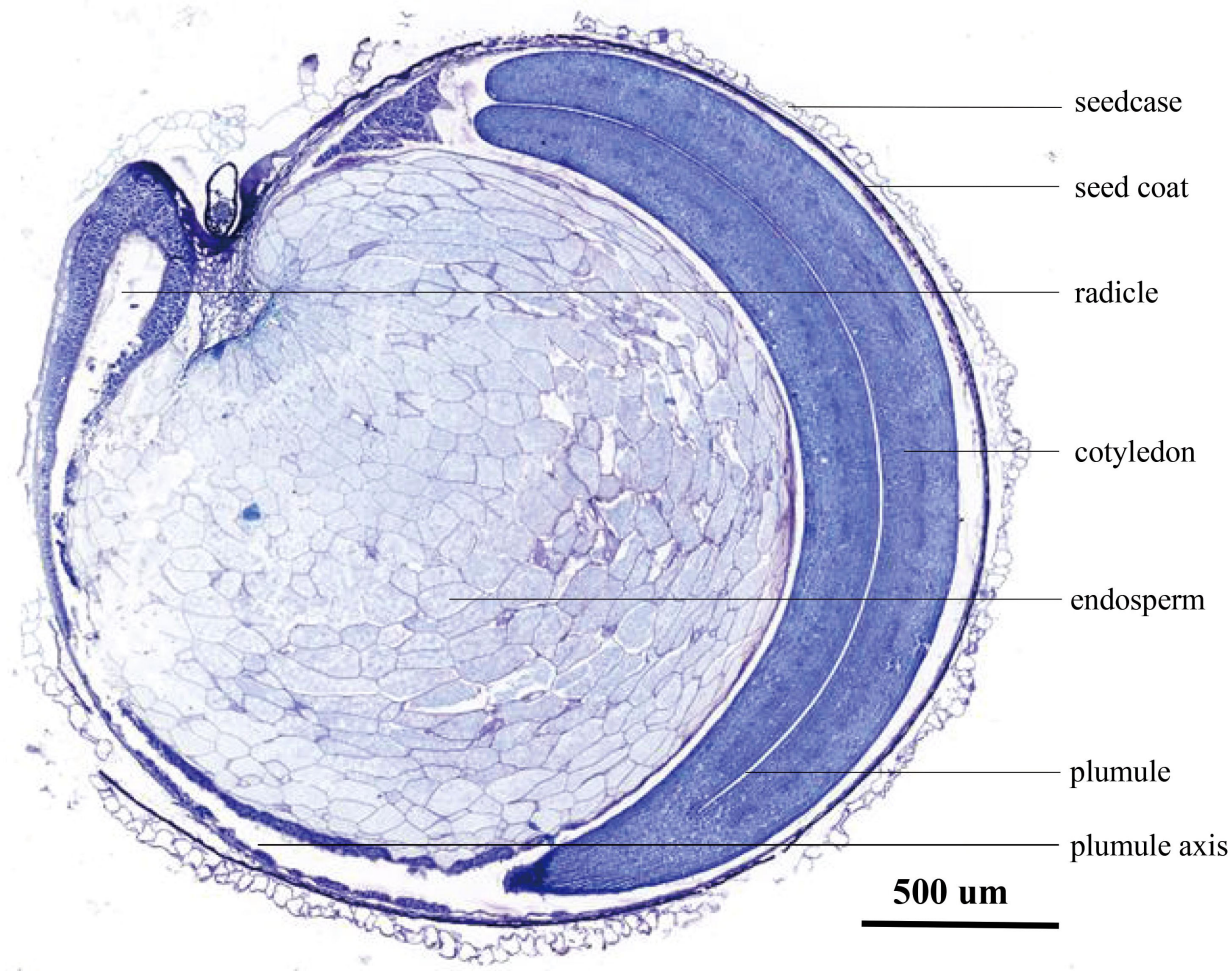
Seed internal structure diagram of Cheng Li No. 2.

4.4 The structural specificity of quinoa seeds may affect the specificity of hormone regulationThe endosperm plays a crucial role in seed germination. During seed water absorption and expansion, diverse enzymes are activated by changes in endogenous hormone levels. These enzymes facilitate the breakdown of energy storage compounds, thereby supplying the essential energy required for embryonic growth (Yan et al., 2014; McGinty et al., 2021). The paraffin sections reveal significant differences in seed structure between quinoa, a distinct heterotetraploid plant variety, and conventional crop seeds such as rice, wheat, and corn. The embryos of rice, wheat, and corn are enveloped by the endosperm, while quinoa endosperm is enveloped by the embryos (**Figure 9**). Hence, it is postulated that the dissimilarity in seed structure potentially underlies the susceptibility of quinoa towards PHS.

The seed coat of quinoa is a barrier for the release of endogenous ABA, and the sensitivity of seeds to ABA is influenced by this factor, or they are not sensitive to ABA (Ceccato et al., 2015). The quinoa cultivar “Qaidam Red-1” has brown seed color, and its endogenous ABA content decreases gradually during germination, which is different from “Cheng Li No. 2”, whose seed color is black (Hao et al., 2022). In addition to seed coat structure and seed coat color, the inconsistent germination rates of different quinoa varieties may also be caused by differences in seed inflorescence and perianth (Vilcacundo and Hernández-Ledesma, 2017; Wu et al., 2019; Pan et al., 2023b). Previous studies have indicated that the endogenous hormones ABA, IAA, and JA exert inhibitory effects on seed germination, while GAs, ETH, and BR stimulate seed germination. However, the specific regulatory mechanisms governing the influence of SA and CTK on seed germination necessitate further comprehensive investigation. (Penfield, 2017; Yanmei et al., 2018; Ahammed et al., 2020; Barreto et al., 2020; Xiong et al., 2021; Ali et al., 2022). Our results of this study showed that the alterations in endogenous hormone levels and associated regulatory genes during the germination process of quinoa seeds differ significantly from those observed in conventional crop seeds, except for ETH, BR, CTK, and SA. Following 4-12 hours of seed germination, the levels of endogenous ABA, IAA, and JA, along with the expression levels of numerous documented negative regulatory genes associated with seed dormancy, exhibited a noteworthy increase (*P* < 0.05). Conversely, the expression levels of genes associated with GA_3_ content and GAs synthesis demonstrated a significant decrease (*P* < 0.05).

Plant hormones are closely related to endosperm, and we speculate that the results of hormones such as ABA in quinoa germination may be related to the specific structure of quinoa seeds, a conclusion to be explored in further studies.

## 5 Conclusion

The results of this study offer empirical support for the notion that the hormonal regulation process during quinoa seed germination exhibits distinct characteristics that are species-specific. This discovery holds considerable implications for the cultivation and progress of quinoa cultivars with resistance to PHS, while also providing valuable insights in this research field. This study shows that quinoa seed germination is intricately linked to energy substances, including α-amylase, endogenous hormones, and soluble sugars. By analyzing the regulatory roles of eight different types of plant endogenous hormones during quinoa seed germination, it was found that the levels of various endogenous hormones showed continuous fluctuations during quinoa seed germination, which suggests that the regulation of quinoa seed germination involves the influence of multiple hormones. The network of endogenous hormones regulating seed germination in quinoa is complex and different from traditional crops. The analysis of transcriptome sequencing data unveiled a total of 99 genes that exhibited differential expression in relation to hormone-regulated pathways governing quinoa seed germination. Out of these, 73 genes demonstrated statistically significant differential expression (*P* < 0.05). Among the significant genes, 14 may serve as pivotal elements within the regulatory network governing quinoa seed germination, as they exhibit close associations with this process. Additionally, diverse quinoa varieties exhibited distinct alterations in hormone content, and disparities were also observed in the seed structure of quinoa when compared to conventional crops.

## Data availability statement

The datasets presented in this study can be found in online repositories. The names of the repository/repositories and accession number(s) can be found below: Bioproject accession number: PRJNA1028334.

## Author contributions

FZ: Conceptualization, Data curation, Formal analysis, Methodology, Software, Visualization, Writing – original draft, Writing – review & editing. CZ: Data curation, Software, Validation, Visualization, Writing – review & editing. WG: Data curation, Software, Validation, Visualization, Writing – review & editing. YG: Data curation, Software, Validation, Visualization, Writing – review & editing. XP: Data curation, Software, Validation, Visualization, Writing – review & editing. XY: Formal analysis, Writing – review & editing, Data curation, Software. XW: Formal Analysis, Funding acquisition, Resources, Validation, Visualization, Writing – original draft, Writing – review & editing, Conceptualization, Data curation. YS: Formal analysis, Software, Writing – review & editing, Funding acquisition, Methodology, Resources, Validation, Visualization, Writing – original draft.

## Supplementary material

Supplementary Text 1 The contents of ABA, ZT, IAA, JA, GA_3_, and SA were determined by HPLC-ESI-MS/MS internal standard method.

Supplementary Table 1 List of specific primer sequences.

Supplementary Table 2 96 GO team related to plant hormones.

Supplementary Table 3 366 enrichment pathways of KEGG.

Supplementary Table 4 The 99 gene sequences meet the criteria of |log2 (fold change)| > 1.
