# Supplementary material for: Regulatory function of the endogenous hormone in the germination process of quinoa seeds
Source: Front Plant Sci. 2024 Jan 8;14:1322986. doi: 10.3389/fpls.2023.1322986 (PMC10801742; doi:10.3389/fpls.2023.1322986)
Supplement: Supplementary Text 1 — The contents of ABA, ZT, IAA, JA, GA3, and SA were determined by HPLC-ESI-MS/MS internal standard method. [file DataSheet_1.pdf]

## The ABA, ZT, IAA, JA, $GA_3$, and SA contents was conducted using the HPLC-ESI-MS/MS internal standard method

### 1. Standard solution configuration

(1) Take 988 μl of a methanol solution and introduce it into a 1.5ml centrifuge tube. Proceed to add 500 μg/ml of each hormone standard reserve liquid, 2 μl each, and mix thoroughly. This mixture should be prepared as a mother liquor with a final concentration of 1 μg/ml for future utilization.

(2) Take 988μl of methanol solution and transfer it into a 1.5ml centrifuge tube. Then, add 2 μl of a standard reserve liquid containing each hormone internal standard at a concentration of 500 μg/ml. Thoroughly mix the contents and designate it as the internal standard with a final concentration of 1 μg/ml in the mother liquor, to be utilized for subsequent procedures.

(3) The standard curve was established using a methanol solution, with final concentrations of 0.1 ng/ml, 0.2 ng/ml, 0.5 ng/ml, 2 ng/ml, 5 ng/ml, 20 ng/ml, 50 ng/ml, and 200 ng/ml. Each data point included a 20 ng/ml internal standard curve solution.

### 2. Mobile phase configuration

Organic phase: 900 ml of pure methanol was introduced into a 1 L volumetric bottle for chromatography purposes. Subsequently, 1 ml of formic acid was added, followed by the addition of methanol to reach a total volume of 1 L. The mixture was thoroughly mixed by inverting the bottle.

Inorganic phase: 900 ml of ultra-pure water was introduced into a 1 L volumetric bottle. Subsequently, 1 ml of formic acid was added to achieve a constant volume of 1 L. The mixture was thoroughly mixed by inverting the bottle.

### 3. plant hormone extraction

This experiment builds upon previous methodologies for extracting and measuring plant hormones, incorporating optimization and improvements (Niu et al., 2014; Liao et al., 2021). The samples, stored at -80°C, were rapidly pulverized into powder using liquid nitrogen. Subsequently, the samples were precisely weighed in test tubes and supplemented with 10 ml of acetonitrile solution and 8 μl of internal standard mother liquor. The samples were subjected to overnight extraction at 4°C, followed by centrifugation at 4°C and 12000 g for 5 minutes to obtain the supernatant. Following precipitation, a 5 ml solution of acetonitrile was reintroduced, and the resulting supernatant was subjected to two extractions. To purify the impurities, an adequate quantity of C18 and GCB were added. The supernatant was then subjected to centrifugation at 4°C and 12000 g for 5 minutes, after which it was collected and dried using nitrogen. The dried sample was subsequently dissolved in 400 μl of methanol, filtered through a 0.22 μm organic phase membrane, and stored in a -20°C refrigerator for

machine detection.

**4. Plant hormone test conditions**

The chromatographic column utilized in the study was a Poroshell 120 SB-C18 reverse-phase column with dimensions of 2.1× 150 and a particle size of 2.7 um. The column temperature was maintained at 30℃. The mobile phase consisted of a mixture of methanol and 0.1% formic acid (A) and water with 0.1% formic acid (B). An injection volume of 2μL was used. The elution gradient was determined according to Table 1.

Table1． Gradient Parameters of HPLC

| Time(min) | Flow rate (mL/min) | A% |
|---|---|---|
| 0-1 | 0.3 | 20 |
| 1-3 | 0.3 | increment from 20 to 50 |
| 3-9 | 0.3 | increment from 50 to 80 |
| 9-10.5 | 0.3 | 80 |
| 10.5-10.6 | 0.3 | decrease from 80 to 20 |
| 10.6-13.5 | 0.3 | 20 |

Mass spectrum parameters included the ionization mode, which was ESI positive and negative ion mode respectively monitoring. The scanning type employed was MRM. The air curtain gas pressure was set at 15 psi. The spray voltage was +4500 V for positive ion mode and -4000 V for negative ion mode. The atomizing gas pressure was 65 psi, while the auxiliary gas pressure was 70 psi. The atomization temperature was maintained at 400℃, as indicated in Table 2.

Table2. selected reaction monitoring conditions for protonated or deprotonated plant hormones ([M+H] + or [M-H]-)

| Name | Polar | Parent ion (m/z) | Daughter ion (m/z) | Cluster Voltage (V) | Collision energy (V) |
|---|---|---|---|---|---|
| IAA | + | 176.1 | 129.8*/102.9 | 65 | 12/42 |
| ABA | - | 263.1 | 153.1*/204.2 | -60 | -14/-27 |

| Name | Polar | Parent ion (m/z) | Daughter ion (m/z) | Cluster Voltage (V) | Collision energy (V) |
| --- | --- | --- | --- | --- | --- |
| $GA_3$ | - | 345.2 | 143.0*/239.2 | -80 | -30/-33 |
| ZT | + | 220.3 | 147.9*/202.1 | 92 | 22/16 |
| SA | - | 137 | 92.9*/65 | -50 | -20/-39 |
| JA | - | 209.2 | 59.1* | -54 | -16 |

Note: The ions marked with * are quantitative ions.

**5. Data analysis**

In this experiment, multiple fragment ions were detected, and their peak time and response value ratio were compared to a standard product to determine the identity of the detected object. The internal standard quantitative method was employed, wherein a known quantity of the internal standard sample was added to the standard sample to create a mixed standard sample. Additionally, a series of working standard samples with known concentrations were prepared. The mole ratio between the mixed standard sample and the internal standard sample remained constant. The response value of the chromatographic column was determined by injecting (standard sample peak area/internal standard sample peak area). By establishing a linear relationship between the response value and the concentration of the working standard sample, the linear regression equation of the standard curve was derived. To determine the concentration of the component to be measured, a known quantity of the internal standard sample was added to the unknown sample and injected into the chromatographic column.


Liao, X., Hong, Y., and Chen, Z. (2021). Identification and quantification of the bioactive components in Osmanthus fragrans roots by HPLC-MS/MS. *Journal of Pharmaceutical Analysis* 11(3)**,** 299-307. doi: 10.1016/j.jpha.2020.06.010.

Niu, Q., Zong, Y., Qian, M., Yang, F., and Teng, Y. (2014). Simultaneous quantitative determination of major plant hormones in pear flowers and fruit by UPLC/ESI-MS/MS. *Anal. Methods* 6(6)**,** 1766-1773. doi: 10.1039/c3ay41885e.
